# A female signal reflects MHC genotype in a social primate

**DOI:** 10.1186/1471-2148-10-96

**Published:** 2010-04-07

**Authors:** Elise Huchard, Michel Raymond, Julio Benavides, Harry Marshall, Leslie A Knapp, Guy Cowlishaw

**Affiliations:** 1 Institut des Sciences de l'Evolution, Université Montpellier 2, Place Eugène Bataillon, CC 065, 34 095 Montpellier cedex 05, France; 2 CNRS-UMR5554, Place Eugène Bataillon, CC 065, 34 095 Montpellier cedex 05, France; 3 Department of Behavioural Ecology and Sociobiology, Deutsches Primatenzentrum, Kellnerweg 4, 37077 Göttingen, Germany; 4 Institute of Zoology, Zoological Society of London, Regent's Park, London NW1 4RY, UK; 5 Department of Biological Anthropology, University of Cambridge, Downing Street, Cambridge CB2 3DZ, UK

## Abstract

**Background:**

Males from many species are believed to advertise their genetic quality through striking ornaments that attract mates. Yet the connections between signal expression, body condition and the genes associated with individual quality are rarely elucidated. This is particularly problematic for the signals of females in species with conventional sex roles, whose evolutionary significance has received little attention and is poorly understood. Here we explore these questions in the sexual swellings of female primates, which are among the most conspicuous of mammalian sexual signals and highly variable in size, shape and colour. We investigated the relationships between two components of sexual swellings (size and shape), body condition, and genes of the Major Histocompatibility Complex (MHC) in a wild baboon population (*Papio ursinus*) where males prefer large swellings.

**Results:**

Although there was no effect of MHC diversity on the sexual swelling components, one specific MHC supertype (S1) was associated with poor body condition together with swellings of small size and a particular shape. The variation in swelling characteristics linked with the possession of supertype S1 appeared to be partially mediated by body condition and remained detectable when taking into account the possession of other supertypes.

**Conclusions:**

These findings suggest a pathway from immunity genes to sexual signals via physical condition for the first time in females. They further indicate that mechanisms of sexual selection traditionally assigned to males can also operate in females.

## Background

Extravagant phenotypic traits are widespread in males and largely assumed to be selected by female preferences. Indeed, decades of work suggest that males in good condition indicate their genetic quality through the production of costly ornaments [e.g. [1-3]]. As a result, choosy females may gain indirect fitness benefits by transmitting "good genes" to their offspring [reviewed in [1]].

In contrast, the evolution of female ornaments is poorly understood, especially in species displaying conventional sex roles (i.e. where intrasexual competition is most intense in males and parental care mostly undertaken by females) [4-6]. Recent evidence suggests that exaggerated phenotypic traits in females could be selected by male mate choice [guppies: [7], barn owls: [8]]. Indeed, theory predicts that mutual mate choice can evolve in species with conventional sex roles whenever males invest in offspring, pay high costs to breed (limiting the number of breeding attempts in a lifetime) or low costs to find a mate, or when female quality is highly variable [9-11].

However, the nature of the benefits obtained by choosy males remains unclear. Although females that invest in costly ornaments might be less able to invest in reproduction [12,13], some studies suggest that female ornaments can reliably indicate direct benefits in the form of fecundity [insects: [14], reptiles: [15], birds: [16,17]]. Nevertheless, the possibility that female signals further advertise indirect benefits such as good genes remains poorly explored from an empirical perspective [but see [18,19]].

Theoretical and empirical arguments suggest that genes of the Major Histocompatibility Complex (MHC) may influence ornament expression [20]. MHC molecules play a crucial role in the vertebrate immune system by presenting specific antigens to immunocompetent cells. The hypothesised mechanism linking MHC genes to the expression of condition-dependent ornaments follows the original framework proposed by Hamilton and Zuk [21]: particular MHC genes can provide resistance to severe pathogenic pressures, enabling individuals possessing such genes to afford the production of costly ornaments. In line with this, associations between the expression of condition-dependent male ornaments and specific MHC genotypes have already been reported [ring-necked pheasants: [22], white-tailed deer: [23]]. Similarly, individuals possessing a high MHC diversity (i.e. the number of distinct MHC alleles) may be more efficient at fighting a wider range of pathogens [24]. It is therefore also possible that MHC diversity positively influences ornament expression, although there is no direct evidence to support this hypothesis.

**Figure 1:**
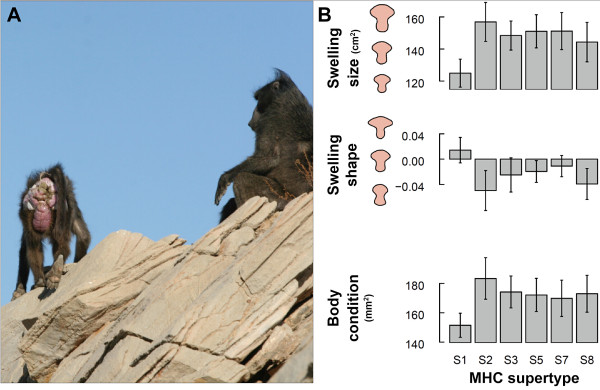
**MHC-associated variation in sexual swelling components and body condition**. (A) A fully-swollen female baboon mate-guarded by an adult male. (B) Effect of six MHC supertypes on swelling components and body condition (note that the results relative to MHC supertype S11 are not displayed because S11 is systematically possessed in association with S2 - see Table S1, Additional File 1). Means and standard errors are shown. The variation in swelling area or swelling shape estimator is illustrated on the relevant axis.

The present study investigates the links between a female sexual signal, MHC genotype, and body condition in a species characterized by conventional sex roles. Our focus is on sexual swellings in a wild population of desert baboons (*Papio ursinus*). Female baboons, like many primates, show cyclical changes in the size, shape, and colour of the anogenital skin (Fig 1A). These perineal swellings, which are associated with sexual receptivity [25,26], reach maximal size around ovulation [27] and attract males independently of olfactory or behavioural cues [28]. Although sexual swellings have been proposed to reliably indicate female quality [29,30], this has long proven difficult to demonstrate [31,32]. Nevertheless, recent work suggests that in this population, at least, sexual swellings may indicate quality: high-condition females produce larger swellings, and these are preferred by males [33]. We have also found that inter-individual differences in the size as well as in the shape of sexual swellings are maintained across consecutive cycles [33,34]. Here we ask (1) whether the size or shape of sexual swellings might advertise genetic quality, indexed by both MHC genotype and diversity, and (2) whether the effect of MHC on sexual swellings might be mediated by physical condition. Our results suggest that sexual swelling size and shape reflect MHC genotype, and that MHC-associated variation in these swelling components may be partially mediated by body condition.

## Methods

### Individual data

Data were collected from wild chacma baboons living at Tsaobis Leopard Park, Namibia [for details on the site, see [35]]. Fifteen females from two habituated troops (32 and 57 individuals) were followed over 1-3 oestrous cycles during two consecutive field seasons (July - December 2005, May 2006 - January 2007), producing a total of 25 cycles. Fourteen of these females (7 multiparous, 3 primiparous, and 4 nulliparous) were captured once or twice during the study period (captures occurred in July 2005 and October 2006), to gather biological samples and morphological measurements. These 14 females (24 cycles) constitute the focus of this study. Briefly, troops were captured using individual cages baited with maize and set-up at dusk. The baboons were captured at dawn, anaesthetized using tiletamine-zolazepam, and all processed within a day, in order to be released together the following morning when fully awake.

During capture, age was estimated by dental eruption and wear [33] and body size was measured as the crown-rump length. Body condition was indexed by the standard anthropometric measure of cross-sectional subcutaneous fat, the Mid-Upper Arm cross-sectional Fat (MUAF), obtained by combining the arm circumference C with the triceps skinfold S using [36]. MUAF indices were available for all 14 females, including repeated measures for four females from different years (2005-2006), to test for MHC-associated variation in condition. We tested for MHC-associated variation in swelling characteristics using only those females for which MUAF indices were available within four months of cycling (13 females and 20 cycles). The remaining female was captured more than a year after cycling. In this subsample, the time elapsed between our quantification of swelling size and shape (i.e. the day of maximal swelling size) and the measurement of MUAF (i.e. the day of capture) was on average 52.4 days (SD = 34.9, range = 2-105 days). Because the upper range of the time elapsed between our MUAF measurements and sexual swelling quantification is long, we investigated the temporal variation in MUAF scores in ten females that had been captured on at least two occasions (this sample included six females of the same study groups that had not cycled but had been captured repeatedly during the study). MUAF measurements taken in October 2006 show a mean (± sd) of 30.1 ± 13.6% difference compared to those taken in July 2005, so the time elapsed between MUAF scores and sexual swelling quantification does represent a source of error in our analysis. However, MUAF measurements were also significantly correlated across years within females (Spearman's correlation: r_s _= 0.67, n = 10, *P *= 0.04), suggesting that females consistently differed in their body condition over time (individual means: range = 129.0 - 235.4 mm^2^, mean ± SD = 169.7 ± 32.1 mm^2^, n = 14 females).

Dominance ranks were calculated from behavioural interactions across the study period [33]. In order to control for differences in troop size, a female's rank was expressed as the percentage of adult females ranking below her.

### Swelling size and shape

Sexual swellings were measured according to two characteristics, their size and shape, at the maximally swollen period for each female cycle. This period was assessed visually by comparing high-resolution digital photographs taken throughout the cycle (using a Canon Eos 20D camera) from behind the female (posterior) and from her side. An average of 47 posterior photographs was taken per female cycle (within the same day of the maximally swollen period) using a wide range of distances (8-40 m). Three to five suitable photos (i.e. with minimal angle and no obstruction by the tail) per cycle were selected for subsequent analysis. The contour (two-dimensional outline) of the swelling was manually extracted using a semi-automatic image processor implemented in Matlab 7.0. to estimate size and shape.

The size of the sexual swelling was measured as the area of the swelling contour, scaled either by telemetry [11 females, 21 cycles: see 33 for full details] or by using direct measurements of tail width and ischial callosities taken during capture (3 females, 3 cycles). Maximal swelling area was then estimated for each cycle of each female by averaging across contours. Swelling size estimates showed considerable variability across females (mean ± SD and range, for individual means across cycles: 150.5 ± 32.7 cm^2^, 95.6 - 194.8 cm^2^, n = 14 females). The swelling size estimates obtained through telemetry and relative measurements respectively ranged within 4% and 7% of direct swelling measurements (ascertained through a comparison made for four females that were swollen during capture).

The shape of the sexual swelling was quantitatively measured using a method based on elliptic Fourier descriptors. Briefly, coordinate information describing the swelling contour was transformed into Fourier coefficients using the SHAPE software [37]. Fourier coefficients were subsequently normalized to be invariant with respect to the size and rotation of the contour. Principal component analysis (PCA) was then carried out on these coefficients. The score of a swelling contour on the first component was used as a quantitative shape estimator for each sexual swelling contour and averaged across pictures taken during the same cycle of the same female. The resulting swelling shape scores were variable across females (mean ± SD and range, for individual means across cycles: -0.02 ± 0.07, -0.17 - 0.08, n = 14 females) but not significantly correlated with swelling area (Spearman's correlation: r_s _= -0.23, n = 14, *P *= 0.43). Full details regarding swelling shape estimation are provided in Huchard et al. [34].

### MHC typing and supertype classification

Baboons across six troops (n = 199), including the 14 females under study, were genotyped at the highly polymorphic *Mhc-DRB *exon 2, including the entire antigen-binding region, using tissue samples collected during capture. Twenty-three distinct *Mhc*-*DRB *sequences (Genbank accession numbers DQ339722-DQ339737 and EU244816-EU244822) were identified. These were non-randomly associated within individuals, defining haplotypes. Fifteen haplotype configurations were identified, each carried by 1 (0.025%) to 52 (26%) individuals and comprising 1-4 *Mhc-DRB *sequences so that each individual carried 2-8 *Mhc-DRB *sequences (Mean ± SD = 5.15 ± 1.47). Details regarding genotyping are provided elsewhere [38,39]. MHC molecules binding similar antigens can be grouped into a supertype [40]. The biological relevance of such classification is supported by a growing body of evidence [e.g. [41-43]]. We classified baboon *Mhc-DRB *sequences into 12 discrete supertypes on the basis of the physico-chemical amino-acid properties of these nucleotides that were positively selected, and thus assumed to be involved in antigen processing by the MHC molecule. Full details regarding supertype classification are provided in Huchard et al. [39]. MHC individual diversity is calculated here as the total number of distinct *Mhc-DRB *supertypes, which is highly correlated to the total number of *Mhc-DRB *sequences (Spearman's correlation: r_s _= 0.90, n = 199, *P *< 10^-3^). The choice of using *Mhc-DRB *supertypes (rather than sequences or haplotypes) is here justified by both statistical and biological arguments:

(1) Our small sample size precludes the analysis of sequence- or haplotype-specific effects because most of them are possessed by only a few females: a specific sequence is possessed by an average of 2.9 females (sd = 2.6, median = 2), and a specific haplotype is possessed by an average of 1.7 females (sd = 2.3, median = 1) in our sample. In contrast, a specific supertype is possessed by 5.4 females on average (sd = 3.4, median = 6).

(2) The detection of sequence-specific effects in such conditions is further complicated by linkage disequilibrium (i.e. non-random association of sequences into haplotypes). Reciprocally, haplotype-specific effects may be hardly detectable due to specific, possibly conflicting effects of the linked sequences which constitute the haplotype.

(3) In contrast, pathogen-driven selective pressures should act similarly on MHC sequences possessing similar binding affinities. The detection of supertype-specific effects on phenotypic traits might thus be possible if some supertypes confer protection or susceptibility to a current pathogenic pressure.

The females involved in this study possessed 2-7 (mean ± SD: 4.4 ± 1.5) MHC supertypes.

### Statistical analyses

We analysed the effects of MHC genotype and body condition on the size and shape of maximal swellings using Linear Mixed Models (LMMs). We also considered the effects of female age, dominance rank, group membership, and body size on sexual swellings [29,31,33]. Age and body size are correlated in this sample (Spearman's correlation: r_s _= 0.66, n = 14, *P *= 0.01) and so were not introduced together in the same model. All LMMs explaining swelling size and shape therefore included three control variables fitted as fixed factors: dominance rank, group membership, and age. Including body size instead of age in the models did not affect the significance of the results (data not shown). Female identity was fitted as a random factor, to control for repeated measures of swelling size and shape (due to repeated cycles) and body condition (due to repeated captures) within females.

The possession of an MHC supertype was coded as a binary factor (presence or absence). The association between an MHC supertype and swelling components was tested for seven (out of 12) supertypes (denoted S1, S2, S3, S5, S7, S8 and S11) which were possessed by more than three females, to avoid statistical inferences based on a minimal number of individuals. The possession of these supertypes was not always statistically independent from the possession of the others (see Additional file 1, Table S1, for details). In particular, the possession of S11 was systematically associated with the possession of S2, so that only S2 was used in our analyses. The effect of each genetic parameter (i.e. individual MHC diversity and each of the seven supertypes) on swelling size and shape was thus first tested by introducing this parameter individually (one at a time) as a fixed factor into a model containing the control variables described above. As a result, each model contained a maximum of four fixed factors (three control variables plus one parameter tested) and one random factor.

To analyse the effect of body condition on swelling size and shape, the MUAF index was similarly introduced as a fixed factor into a model containing the control variables described above (and in the absence of genetic parameters). The MUAF score for each female was always taken within four months of a given cycle.

We further investigated the effect of genetic parameters on body condition using the same procedures. MUAF was modelled as a function of female age, dominance rank, and group membership plus the considered genetic parameter. Female identity was fitted as a random factor to account for repeated measures of condition within females.

After identifying a genetic parameter with a significant effect on both swelling characteristics and body condition (MHC supertype S1), we introduced it together with the MUAF scores in two models respectively explaining swelling size and shape (with the same structure of fixed and random effects described above) to determine whether the effect of MHC supertype S1 on swelling components could be entirely or partially mediated by MUAF scores (i.e. if the S1 effect disappeared in the presence of the MUAF scores, or the MUAF effect disappeared in the presence of S1, respectively).

Finally, because all our study subjects possess several supertypes with potentially contradicting effects on phenotypic traits, we investigated whether the apparently deleterious effect of S1 was still detectable in the presence of other supertype effects (See Additional file 1, Tables S10-S12, for details on the statistical methods and results).

The significance of the fixed factors was evaluated using F-tests calculated according to the principle of marginality, testing each term after all others (i.e. comparing two models differing only in the presence of the tested fixed effect) [44]. Inferences were drawn from the full models (all predictors present) [45] and also after model simplification to confirm the stability of the models. Model simplification was performed using a classical backward selection procedure, by removing the variable showing the highest *P *value (when *P *> 0.05) from the model to select a best-fit set of explanatory variables [46]. The full and simplified models gave the same results, which are only reported for the full models in the main text (the simplified models are reported in Additional file 1). All statistical analyses were carried out using software R 2.8.0 (R Development Core Team, 2003).

## Results

Individual MHC diversity (number of MHC supertypes) was not related to swelling size or shape (Table 1). Nevertheless, one MHC supertype (S1) was associated negatively with swelling size and positively with the swelling shape score (Table 1, Fig 1B). As a result, females carrying S1 had smaller swellings with higher scoring shapes (mean ± SD: swelling area = 125.0 ± 23.1 cm^2^, swelling shape score = 0.01 ± 0.05, n = 7) than other females (swelling area = 176.0 ± 16.4 cm^2^, swelling shape score = -0.05 ± 0.07, n = 7).

**Table 1:** Results of the linear mixed-effect models testing MHC-associated and condition-dependent variation in swelling size and shape, as well as MHC-associated variation in body condition.

Variable	Swelling size				Swelling shape				Body condition			
	
	Est. [SD]	F_1, df_	df	*P*	Est. [SD]	F_1, df_	df	*P*	Est. [SD]	F_1, df_	df	*P*
Age^1^	-1.54 [2.34]	0.43	10	0.52	-1.4 × 10^-2 ^[3.4 × 10^-3^]	17.5	10	< 0.01	-1.37 [3.22]	0.18	11	0.70
Social rank^1^	32.57 [28.23]	1.33	10	0.28	7.9 × 10^-2 ^[3.9 × 10^-2^]	7.33	10	0.07	20.79 [34.32]	0.37	11	0.56
Group^1,2^	25.04 [18.02]	1.93	10	0.19	-4.9 × 10^-3 ^[2.5 × 10^-2^]	0.04	10	0.85	-3.66 [23.78]	0.02	11	0.88

MHC diversity^3^	-6.62 [6.66]	0.99	9	0.35	7.1 × 10^-3 ^[1.0 × 10^-2^]	0.49	9	0.50	8.20 [8.72]	0.88	10	0.37
S1^3,4^	-45.90 [14.74]	10.8	9	0.01	8.6 × 10^-2 ^[1.1 × 10^-2^]	64.6	9	< 10^-3^	-54.89 [19.79]	7.70	10	0.02
S2, S11^3,5^	19.49 [17.36]	1.26	9	0.29	-2.7 × 10^-2 ^[2.5 × 10^-2^]	1.22	9	0.29	38.48 [21.19]	2.96	10	0.12
S3^3^	-5.09 [19.83]	0.04	9	0.84	1.2 × 10^-2 ^[2.7 × 10^-2^]	0.18	9	0.68	18.28 [23.30]	0.61	10	0.45
S5^3^	-5.27 [19.53]	0.07	9	0.79	-1.7 × 10^-2 ^[2.7 × 10^-2^]	0.38	9	0.55	9.57 [26.41]	0.13	10	0.72
S7^3^	-3.22 [18.96]	0.03	9	0.89	-1.5 × 10^-2 ^[2.7 × 10^-2^]	0.31	9	0.59	-0.33 [26.04]	0.00	10	0.99
S8^3^	12.23 [21.71]	0.32	9	0.59	-2.6 × 10^-2 ^[3.0 × 10^-2^]	0.73	9	0.41	25.92 [28.34]	0.84	10	0.38
MUAF^3^	0.50 [0.21]	5.53	8	0.05	-6.6 × 10^-4 ^[2.8 × 10^-4^]	5.68	8	0.04	n/a	n/a	n/a	n/a

^1 ^Parameters and tests for control variables (age, social rank, group) are calculated here in the absence of the test variables. See Additional file 1 for the parameters and tests calculated from the full models including either S1 or MUAF.

^2 ^Reference category: smaller troop.

^3 ^Each of the test variables was tested independently (i.e. one test parameter per model), such that the results presented here are a summary of eight different models (in this case the full models, i.e. all predictors present). The individual models testing the influence of MHC supertype S1 are presented in Additional file 1 (see Tables S2-S3 for the models explaining swelling characteristics, and Tables S8-S9 for the models explaining body condition), as well as the models testing the influence of MUAF on swelling characteristics (Tables S4-S5).

^4 ^Phenotypic effects of S1 on swelling components remain significant after Bonferroni corrections for multiple tests, with a significance threshold α = 0.0125 for a total of four independent tests involving MHC supertypes S1, S3, S5 and S8 (the possession of S2 and S7 is non-independent from the possession of several other supertypes: see Table S1 in Additional file 1). In contrast, the effects of MHC supertype S1 on body condition is reduced to a trend (*P *= 0.08) after correction for multiple testing.

^5 ^Supertypes S2 and S11 are always possessed together.

We then explored the interconnections between swelling components, supertype S1, and body condition. Body condition was linked to swelling size by a positive relationship and to swelling shape by a negative relationship (Table 1). According to these results, females in good condition tend to produce large swellings with a low scoring shape. In addition, S1 was negatively associated with body condition (Figure 1B, Table 1). Females possessing S1 were thus in lower condition (mean MUAF index ± SD: 156.6 ± 23.4 mm^2^, n = 7 females) than other females (194.4 ± 33.2 mm^2^, n = 7 females). When S1 and body condition are introduced together in a model explaining either swelling size or shape, the influence of condition disappears (see Tables S6-S7 in Additional file 1), suggesting that condition at least partially mediates the genetic effects on this ornament.

We further explored whether the relationships between supertype S1 and phenotype may be confounded by the fact that individuals have multiple supertypes. We introduced S1 together with other (non-colinear) supertypes in three models explaining each of the three phenotypic traits of interest (swelling size, swelling shape, and body condition) to check for the consistency in the direction and strength of S1 effects in the presence of other supertypes. The phenotypic variations associated with the possession of S1 appear largely maintained in the presence of other supertypes: the directionality and strength of the S1 effects is consistent, and significance is reached in most models despite their overparameterisation (See Additional file 1, Tables S10-12). This suggests that the apparently deleterious effect of S1 remains detectable despite the potentially conflicting effects of the other supertypes possessed by individuals.

Finally, since S1 currently appears disadvantageous, we investigated circumstantial evidence for counter selection. Across the last two generations, the frequency of S1 has declined from 34% in adult baboons (age > 8 years, n = 78) to 24% in juveniles (age < 3 years, n = 72) (Chi-square unilateral test: χ^2 ^= 3.0, *P *= 0.04). This represents the greatest decrease recorded among the 12 supertypes, although it should be noted that at least one supertype showed a frequency increase of similar amplitude (proportional change in S1: -0.10; all other supertypes: -0.05 to 0.10). These fluctuations in supertype frequencies should thus be interpreted with caution.

## Discussion

Our study reports an association between a specific MHC genotype and the expression of a female sexual signal. MHC supertype S1 is associated with poor female body condition, as well as small sexual swellings with a particular shape, in a wild primate population. Here we consider in more detail the relationship between body condition and signal expression, the advertisement of MHC genes, and the evolution of female signalling in conventional sex roles.

### Female condition and signal expression

Two components of the signal under study, namely swelling size and swelling shape, were found to reflect female body condition, indexed by the mid-upper arm cross-sectional fat MUAF. This result is consistent with the hypothesis that sexual swellings advertise female quality [29,30,33], a pattern also reported for male sexual signals in a variety of species [e.g. [1,47]]. This relationship is further supported by the finding that swelling size is significantly associated with another measure of female body condition in this population, namely residual body mass (i.e. the residuals of a body mass - size regression) [33]. However, a third body condition measure, the Body Mass Index (BMI, calculated as the ratio of body mass over body size^2^) was found to have no effect on baboon swelling shape [34]. While it is reassuring to find that different measures of condition can show the same pattern with signal expression, we should not be surprised when they do not. This is for two reasons. First, and most importantly, different indices reflect different aspects of condition, either individually or in combination, such as fat reserves, muscle mass, and skeletal weight. A signaller will only advertise that element of condition that is important to the receiver, so an effect of 'condition' will only be detected when it is appropriately measured. Second, in this particular instance, it is well-known that ratio measures of condition such as BMI have poor statistical properties and are weak measures of condition [e.g. [48]]. In contrast, mid-upper arm circumference measurements have previously been shown to reflect the body condition of wild baboons [49], and MUAF has been found to predict nutritional status more sensitively than either of its constituent components, i.e. upper arm circumference or triceps skinfold thickness, alone [50].

While we are confident that the associations described between female condition and signal expression are robust, we would still emphasise caution in the interpretation of the results regarding swelling shape. Although we have previously found a male preference for large swellings [33], it is unknown whether males show a comparable preference for swelling shape. Nevertheless, it is notable that female age is associated with shape in the same way as female condition (i.e. older females and females in better condition exhibit more negative shape scores). Male primates consistently express preferences for mature females [51,52], who typically display higher fertility [e.g. [53,54]] and infant survival [e.g. [54,55]] than younger females. A male preference for females with negative scoring swelling shapes would thus provide them with fertility benefits in addition to good genes for their offspring (through MHC and condition-dependent signalling).

### MHC, good genes, and bad genes

Although no association between MHC diversity and the expression of sexual swellings could be detected, the possession of one MHC supertype (S1) is linked with a small and 'unshapely' swelling, together with low body condition. These results suggest a pathway from immunity genes to sexual signals via physical condition. As a result, male baboons may choose mates by scrutiny of condition-dependent ornaments because these signals reveal advantageous, or disadvantageous, MHC genotypes. A similar argument has been made for female choice on the basis of male signals in ring-necked pheasants [56] and white-tailed deer [23]. Since the fitness advantages provided by specific MHC genotypes can depend on fluctuations in pathogen communities and host-parasite coadaptational cycles [e.g. [57]], these results support the Hamilton and Zuk hypothesis [21]: exaggerated ornaments constantly reveal the most successful genotype*environment combinations. According to this hypothesis, sexual selection reinforces natural selection in favouring advantageous alleles or counter-selecting disadvantageous alleles. This may explain the apparent decline observed in the frequency of the supertype S1 in our population.

Interestingly, the association between supertype S1 on swelling components and body condition is negative, suggesting that sexual signals may advertise bad genes. While this may appear surprising at first sight, MHC-associated susceptibility to immune or infectious diseases has been reported as often as MHC-associated resistance in natural populations [58]. The possession of specific MHC supertypes has for instance been associated with high viral loads in HIV in humans [43], or with higher susceptibility to particular gut parasites in lemurs [41], although the immunological mechanism underlying such associations is poorly understood. Pathogen evasion models predict that the recent escape of a pathogen to a given MHC allele or supertype may result in susceptibility to this pathogen by the individuals possessing this genotype [58]. This could occur if individual haplotypes contain non-random combinations of MHC supertypes (i.e. "individual immune repertoires") [39] that complement each other to maximise immune coverage against the set of pathogenic pressures encountered in the environment. The recent escape of a pathogen to a given supertype could thus create a "gap" in any individual immune repertoire that includes this supertype, until selection pressures favour the emergence of new haplotypes through recombination. The negative association reported here, which is apparently not diluted by the possession of other supertypes, might similarly be mediated by an increased susceptibility to a dominant pathogen.

One may wonder whether bad genes would be expected to be advertised in the context of the current "good genes" paradigm. The handicap theory of sexual signalling predicts that the costs of signal production increase for low-condition animals, thus maintaining honesty in signalling [e.g. [1,2]]. Consequently, animals in poor condition are unable to signal their quality through vigorous display because of the costs associated with producing or maintaining such extravagance. Under this hypothesis, the possession of a deleterious allele might be expected to translate into lower condition leading to proportionally higher costs of signal production and an associated poorer signalling performance. Our finding of bad gene advertisement is therefore consistent with the existing framework of honest-signalling models.

### Female sexual signals in conventional sex roles

Finally, and perhaps most importantly, our findings indicate that female sexual signals can advertise heritable aspects of quality just as male ornaments do [e.g. [1]]. Although these results may challenge traditional theories regarding sex roles and sexual signalling, there is growing evidence that the selective pressures favouring the evolution of ornamentation may be of similar nature in both sexes [4,5]. In the case of primates, females may be expected to be choosy because they provide most parental care. However, several other aspects of the mating system of primates living in multimale-multifemale groups can explain the evolution of mutual mate choice. In the case of males, there are potentially high costs associated with reproduction. First, males frequently incur injuries through competition for access to fertile females [59]. Second, once a male has obtained access to a female, mate-guarding 'consortships' place severe constraints on foraging activity [e.g. [60]], and also prevent males from mating with other females. Third, male ability to mate at high rates (and thus with multiple partners) may be limited by physiological constraints, for instance by sperm depletion [61], which may also encourage choosiness. Finally, it appears increasingly plausible that male baboons care for their offspring [e.g. [62-65]] and this probably incurs further costs. In addition to these costs, the potential for male choice is enhanced by the fact that male baboons can freely sample potential mates (due to group living) and female quality is likely to vary extensively, at least in the form of fertility or infant survival that can be socially-mediated or age-related [54,66-72], and also possibly in the form of heritable quality (this study).

Reciprocally, females may benefit from signalling if they compete for mates, which may be the case even in multimale groups of non-seasonally breeding species (where only a few females are typically receptive at the same time) [33]. Females from multimale primate groups are thought to mate multiply to limit the risk of infanticide by non-mated males [73,74] or to predispose multiple males to protect their infants from infanticide by immigrant males [75]. In this context, highly ornamented females would benefit from attracting more partners, or from attracting "key" partners such as the alpha male of the group. Indeed, failure to mate with this male would not only lead to the loss of several possible direct and indirect benefits (e.g. good genes, infant protection services) but also increase the risk of subsequent infanticidal attacks on the offspring he has not sired.

Finally, it might appear difficult to understand why males would choose females who invest resources into costly signals of genetic quality rather than directly into offspring [e.g. [13,54]]. This apparent paradox may be resolved in at least two ways. First, variation in swelling size within and between cycles of the same female are linked to hormonal variation that is believed to reflect fertility [chimpanzes: [76,77], baboons: [78,79]]. As a result, males choosing females with large swellings may be rewarded by direct benefits (fertility) in addition to indirect benefits (good genes for offspring) [33]. Second, the costs of production and maintenance of sexual swellings are temporally separated from maternity costs, due to the ephemeral nature of swellings. The investment of current resources in swelling production may therefore have minimal impact on the resources available for future gestation and lactation. It is even possible that some of the metabolites necessary for swelling production, such as structural macromolecules [e.g. collagen or glycosaminoglycans: [80,81]], are "stored" in swellings and subsequently re-used in other metabolic tasks. The expression of costly ornaments may thus reliably indicate the amount of resources that females are able to secure for future reproduction.

## Conclusions

Our findings indicate, for the first time, MHC-associated variation in the expression of female ornaments, the sexual swellings of wild baboons. Females are not necessarily expected to reveal their genetic quality through costly condition-dependent ornaments in species that display conventional sex roles. Yet, the evolution of male preferences for females signalling good genes might, like in baboons, be favoured by particular aspects of a mating system, and this may affect a broad range of group-living species. Overall, these results do not undermine our global understanding of the evolutionary mechanisms involved in sexual selection, but clearly indicate that the distribution of sex differences is more complex than traditionally proposed [4].

## Authors' contributions

EH designed the study, collected the field data, carried out the molecular genetic studies, performed the statistical analyses and drafted the manuscript. JB analysed the swelling pictures. HM calculated the body condition indices. LAK participated in the design of the study and provided help and logistical support to carry out the molecular genetics. MR and GC participated in the design of the study, provided help and logistical support with data collection and analyses, and helped to draft the manuscript. All authors read and approved the final manuscript.

## Supplementary Material

Additional file 1 Supplementary tables detailing supporting analyses. Click here for file
