# Supplementary material for: A female signal reflects MHC genotype in a social primate
Source: BMC Evol Biol. 2010 Apr 7;10:96. doi: 10.1186/1471-2148-10-96 (PMC2858743; doi:10.1186/1471-2148-10-96)
Supplement: Additional file 1 — Supplementary tables detailing supporting analyses. [file 1471-2148-10-96-S1.PDF]

Additional Material file 1

A female signal reflects MHC genotype in a social primate

Elise Huchard[1,2,3,§], Michel Raymond[1,2], Julio Benavides[1,2], Harry Marshall[4], Leslie A Knapp[5], Guy Cowlishaw[4].

[1] Institut des Sciences de l'Evolution, Université Montpellier 2, Place Eugène Bataillon, CC 065, 34 095 Montpellier cedex 05, France
[2] CNRS-UMR5554, Place Eugène Bataillon, CC 065, 34 095 Montpellier cedex 05, France
[3] Department of Behavioural Ecology and Sociobiology, Deutsches Primatenzentrum, Kellnerweg 4, 37077 Göttingen, Germany
[4] Institute of Zoology, Zoological Society of London, Regent's Park, London NW1 4RY, UK
[5] Department of Biological Anthropology, University of Cambridge, Downing Street, Cambridge CB2 3DZ, UK

[§] Corresponding author: ehuchard@gmail.com

Content:

I. Selection of the supertypes tested in the analysis 2
II. MHC supertype S1 and swelling characteristics 3
III. Body condition and swelling characteristics 4
IV. MHC supertype S1 and body condition 6
V. MHC-associated variation of swelling characteristics and body condition: testing the background effects 7

I. Selection of the supertypes tested in the analysis

Table S1. Selection of the supertypes tested in the analysis. Significant *P*-values are reported in italic.

***MHC-DRB* supertype**	**Number of females possessing supertype**	**Correlation matrix for the possession of supertypes (Spearman's correlations, n = 14 females in each case)**												**Number of supertypes statistically associated**
		**S2**		**S3**		**S5**		**S7**		**S8**		**S11**		
		r_s	***P***	r_s	***P***	r_s	***P***	r_s	***P***	r_s	***P***	r_s	***P***	
**S1**	7	-0.29	0.31	0.15	0.61	-0.32	0.27	-0.15	0.61	0.00	1.00	-0.29	0.32	0
**S2**	6			*0.64*	*0.01*	-0.41	0.14	*-0.60*	*0.04*	*0.75*	*0.00*	*1.00*	*0.00*	4
**S3**	9					-0.47	0.10	*-0.60*	*0.04*	0.26	0.37	*0.64*	*0.01*	3
**S4**	2[1]						n/a	n/a	n/a	n/a	n/a	n/a	n/a	n/a
**S5**	10							*0.85*	*0.00*	-0.22	0.43	-0.41	0.14	1
**S6**	2[1]								n/a	n/a	n/a	n/a	n/a	n/a
**S7**	7									-0.34	0.22	*-0.60*	*0.04*	4
**S8**	8											*0.75*	*0.00*	2
**S9**	3[1]												n/a	n/a
**S10**	1[1]													n/a
**S11**	6													4
**S12**	1[1]													n/a

[1] Supertypes that were possessed by less than three females were discarded to avoid statistical inferences based on minimal sample size.

II. MHC supertype S1 and swelling characteristics

The following tables provide the detailed results of the statistic analyses linking MHC supertype S1 to swelling characteristics, providing the outputs of both the full and reduced models. In all cases, the reference category of the variable “Group” is the smaller group.

Table S2. Output of the full models (all predictors present) testing the effect of supertype S1 on swelling size and shape.

Variable	**Swelling size**				**Swelling shape**[1]			
	Estimate ± SD	$F_{1, df}$	df	*P*	Estimate ± SD	$F_{1, df}$	df	*P*
Age	-1.37 ± 1.78	0.59	9	0.46	$-1.46\times10^{-2} \pm 1.18\times10^{-3}$	151.12	9	$< 10^{-4}$
Social rank	6.69 ± 23.64	0.08	9	0.78	$1.31\times10^{-2} \pm 1.62\times10^{-2}$	65.14	9	$< 10^{-4}$
Group	2.32 ± 15.70	0.02	9	0.89	$-3.29\times10^{-3} \pm 1.07\times10^{-2}$	9.38	9	0.01
S1	-45.90 ± 14.74	10.80	9	0.01	$8.61\times10^{-2} \pm 1.10\times10^{-2}$	64.55	9	$< 10^{-4}$

[1] This model cannot be further reduced.

Table S3. Output of the reduced model (using a backward selection procedure) testing the effect of supertype S1 on swelling size. The corresponding model explaining swelling shape could not be simplified (see Table S2).

Variable	**Swelling size**			
	Estimate ± SD	$F_{1, df}$	df	*P*
Age	ns	ns	ns	ns
Social rank	ns	ns	ns	ns
Group	ns	ns	ns	ns
S1	-45.96 ± 11.70	15.43	12	< 0.01

III. Body condition and swelling characteristics

The following tables provide the detailed results of the statistic analyses linking body condition MUAF to swelling characteristics, providing the outputs of both the full and reduced models. In all cases, the reference category of the variable "Group" is the smaller group.

Table S4. Output of the full models (all predictors present) testing the effect of MUAF on swelling size and shape.

Variable	**Swelling size**				**Swelling shape**			
	Estimate ± SD	$F_{1, df}$	df	*P*	Estimate ± SD	$F_{1, df}$	df	*P*
Age	-0.52 ± 2.16	0.06	8	0.82	$-1.48x10^{-2} \pm 3.11x10^{-3}$	22.71	8	<0.01
Social rank	13.24 ± 26.00	0.26	8	0.62	$0.11 \pm 3.61x10^{-2}$	8.64	8	0.02
Group	26.66 ± 16.37	2.65	8	0.14	$-3.72x10^{-3} \pm 2.26x10^{-2}$	0.03	8	0.87
MUAF	0.50 ± 0.21	5.53	8	0.05	$-6.61x10^{-4} \pm 2.78x10^{-4}$	5.68	8	0.04

Table S5. Output of the reduced models (using a backward selection procedure) testing the effect of MUAF on swelling size and shape.

Variable	**Swelling size**				**Swelling shape**			
	Estimate ± SD	$F_{1, df}$	df	*P*	Estimate ± SD	$F_{1, df}$	df	*P*
Age	ns	ns	ns	ns	$-1.50x10^{-2} \pm 2.83x10^{-3}$	27.90	9	$< 10^{-3}$
Social rank	ns	ns	ns	ns	$1.06x10^{-1} \pm 3.43x10^{-2}$	9.56	9	0.01
Group	ns	ns	ns	ns	ns	ns	ns	ns
MUAF	0.51 ± 0.20	6.36	11	0.03	$-6.59x10^{-4} \pm 2.64x10^{-4}$	6.24	9	0.03

Table S6. Output of the full models (all predictors present) testing the effect of MHC supertype S1 together with MUAF on swelling characteristics.

Variable	**Swelling size**				**Swelling shape**[1]			
	Estimate ± SD	$F_{1, df}$	df	*P*	Estimate ± SD	$F_{1, df}$	df	*P*
Age	-0.41 ± 1.79	0.05	7	0.83	$-0.01 \pm 1.30x10^{-3}$	135.58	7	$<10^{-3}$
Social rank	1.13 ± 22.58	0.00	7	0.96	0.13 ± 0.02	65.53	7	10^{-3}
Group	8.25 ± 16.12	0.26	7	0.62	0.03 ± 0.01	7.42	7	0.03
S1	-39.67 ± 18.34	4.68	7	0.07	0.09 ± 0.01	41.58	7	$<10^{-3}$
MUAF	0.23 ± 0.22	1.07	7	0.33	$3.41x\ 10^{-4} \pm 1.60x10^{-4}$	0.04	7	0.84

[1] This model cannot be further reduced.

Table S7. Output of the reduced model (using a backward selection procedure) testing the effect of MHC supertype S1 together with MUAF on swelling size. The corresponding model explaining swelling shape could not be simplified (see Table S6).

Variable	**Swelling size**			
	Estimate ± SD	$F_{1, df}$	df	*P*
Age	ns	ns	ns	ns
Social rank	ns	ns	ns	ns
Group	ns	ns	ns	ns
S1	-44.24 ± 13.88	10.16	10	<0.01
MUAF	0.19 ± 0.18	1.06	10	0.33

IV. MHC supertype S1 and body condition

The following tables provide the detailed results of the statistic analyses linking MHC supertype S1 to body condition MUAF, providing the outputs of both the full and reduced models. In all cases, the reference category of the variable “Group” is the smaller group.

Table S8. Results of the full model (all predictors present) linking MHC supertype S1 to MUAF.

Variable	Estimate ± SD	$F_{1, df}$	df	*P*
Age	-1.54 ± 2.63	0.34	10	0.60
Social rank	-10.59 ± 29.67	0.13	10	0.73
Group	-22.88 ± 20.31	1.27	10	0.29
S1	-54.89 ± 19.79	7.70	10	0.02

Table S9. Output of the reduced model (using a backward selection procedure) testing the effect of supertype S1 on MUAF.

Variable	Estimate ± SD	$F_{1, df}$	df	*P*
Age	ns	ns	ns	ns
Social rank	ns	ns	ns	ns
Group	ns	ns	ns	ns
S1	- 42.21 ± 16.80	6.32	12	0.03

V. MHC-associated variation of swelling characteristics and body condition: testing the background effects

Because all our study subjects possess several supertypes with potentially contradicting effects on phenotypic traits, we investigated whether the apparently deleterious effect of S1 was still detectable in the presence of other supertype effects. The four supertypes which were statistically independent from each other (S1, S3, S5, and S8: see Table S1) were introduced together as fixed factors (in addition to age, rank, and group membership) within the same LMM (still with female identity as a random factor) explaining swelling size (Table S10, first column). The same was also done in two further models explaining swelling shape and body condition (Table S10, second and third columns, respectively). The supertypes S2 and S7 are introduced in separate models (results reported in Tables S11 and S12 respectively) to avoid problems of colinearity, since their possession is not independent from the possession of several other supertypes (Table S1). These analyses are all necessarily compromised by a lack of statistical power (resulting from an overparameterisation of the models), but have been performed to evaluate the consistency in the directionality and strength of the effects of the supertype S1 in the presence of other supertypes. These models have thus not been reduced since this would have led to the drop-out of several supertypes. In all cases, the reference category of the variable "Group" is the smaller group.

Table S10. Output of the full models (all predictors present) testing the effects of the MHC supertypes S1, S3, S5, and S8 (introduced together in the same model) on swelling characteristics and body condition.

Variable	**Swelling size**				**Swelling shape**				**Body condition**			
	Estimate ± SD	$F_{1, df}$	df	*P*	Estimate ± SD	$F_{1, df}$	df	*P*	Estimate ± SD	$F_{1, df}$	df	*P*
Age	-0.55 ± 1.69	0.11	6	0.75	-0.01 ± 1.24x10^{-3}	147.21	6	0	-1.72 ± 3.04	0.31	7	0.61
Social rank	-6.5 ± 26.73	0.06	6	0.82	0.14 ± 0.02	51.66	6	<10^{-3}	-11.49 ± 44.91	0.06	7	0.81
Group	-14.06 ± 18.99	0.55	6	0.49	0.05 ± 0.01	10.65	6	0.02	-30.00 ± 26.43	1.29	7	0.29
**S1**	**-65.36 ± 18.77**	**12.12**	**6**	**0.01**	**0.10 ± 0.01**	**51.73**	**6**	**<10^{-3}**	**-60.66 ± 25.51**	**5.65**	**7**	**0.05**
S3	0.28 ± 16.50	0.00	6	0.99	-6.23x10^{-3} ± 0.01	0.25	6	0.63	26.24 ± 20.89	1.58	7	0.25
S5	-30.03 ± 15.62	3.70	6	0.10	0.01 ± 0.01	1.36	6	0.29	0.14 ± 26.68	0.00	7	0.99
S8	-21.69 ± 18.63	1.36	6	0.29	0.02 ± 0.01	2.07	6	0.2	-6.02 ± 28.46	0.04	7	0.84

Table S11. Output of the full models testing the effects of the MHC supertypes S1 and S2 (introduced together in the same model) on swelling characteristics and body condition.

Variable	**Swelling size**				**Swelling shape**				**Body condition**			
	Estimate±SD	$F_{1, df}$	df	*P*	Estimate±SD	$F_{1, df}$	df	*P*	Estimate±SD	$F_{1, df}$	df	*P*
Age	-1.52±1.92	0.62	8	0.45	$-0.01 \pm 1.24 \times 10^{-3}$	138.85	8	$<10^{-3}$	-2.34±2.82	0.69	9	0.47
Social rank	9.17±25.83	0.13	8	0.73	0.13±0.02	56.25	8	10^{-3}	-1.83±31.73	0.00	9	0.95
Group	4.45±17.39	0.07	8	0.80	0.03±0.02	8.03	8	0.02	-18.84±21.08	0.80	9	0.39
**S1**	**-43.96±17.94**	**6.01**	**8**	**0.04**	**0.09±0.01**	**52.40**	**8**	**$<10^{-3}$**	**-46.88±22.13**	**4.49**	**9**	**0.06**
S2	4.56±15.51	0.09	8	0.78	$1.62 \times 10^{-3} \pm 0.01$	0.03	8	0.88	17.63±20.81	0.72	9	0.42

Table S12. Output of the full models testing the effects of the MHC supertypes S1 and S7 (introduced together in the same model) on swelling characteristics and body condition.

Variable	**Swelling size**				**Swelling shape**				**Body condition**			
	Estimate±SD	$F_{1, df}$	df	*P*	Estimate±SD	$F_{1, df}$	df	*P*	Estimate±SD	$F_{1, df}$	df	*P*
Age	-1.43±1.66	0.74	8	0.41	$-0.01 \pm 1.27 \times 10^{-3}$	143.89	8	$<10^{-3}$	-1.99±2.78	0.51	9	0.53
Social rank	10.92±22.74	0.23	8	0.64	0.03±0.01	5.34	8	0.05	-10.13±30.38	0.11	9	0.75
Group	0.14±14.84	0.00	8	0.99	0.13±0.01	63.45	8	$<10^{-3}$	-25.98±21.35	1.48	9	0.25
**S1**	**-49.50±15.22**	**10.58**	**8**	**0.01**	**0.09±0.01**	**40.50**	**8**	**$<10^{-3}$**	**-58.09±20.86**	**7.75**	**9**	**0.02**
S7	-16.11±13.37	1.45	8	0.26	$8.67 \times 10^{-3} \pm 0.01$	3.83	8	0.09	-13.75±21.53	0.41	9	0.54
